# Type III Interferons: Emerging Roles in Autoimmunity

**DOI:** 10.3389/fimmu.2021.764062

**Published:** 2021-11-26

**Authors:** Sindhu Manivasagam, Robyn S. Klein

**Affiliations:** ^1^ Center for Neuroimmunology & Neuroinfectious Diseases, Washington University School of Medicine, St. Louis, MO, United States; ^2^ Department of Medicine, Washington University School of Medicine, St. Louis, MO, United States; ^3^ Department of Pathology & Immunology, Washington University School of Medicine, St. Louis, MO, United States; ^4^ Department of Neurosciences, Washington University School of Medicine, St. Louis, MO, United States

**Keywords:** interferon lambda, autoimmune diseases, Th1 cells, IFNLR, neuroimmunology and neuropathology

## Abstract

Type III interferons (IFNs) or the lambda IFNs (IFNLs or IFN-λs) are antimicrobial cytokines that play key roles in immune host defense at endothelial and epithelial barriers. IFNLs signal *via* their heterodimeric receptor, comprised of two subunits, IFNLR1 and interleukin (IL)10Rβ, which defines the cellular specificity of the responses to the cytokines. Recent studies show that IFNL signaling regulates CD4+ T cell differentiation, favoring Th1 cells, which has led to the identification of IFNL as a putative therapeutic target for autoimmune diseases. Here, we summarize the IFNL signaling pathways during antimicrobial immunity, IFNL-mediated immunomodulation of both innate and adaptive immune cells, and induction of autoimmunity.

## Introduction

Type III interferons (IFNs) or the lambda IFNs (IFNLs or IFN-λs), generate and sustain antiviral and immunomodulatory cellular responses. Specifically, they are known for their ability to control viral replication and infection at barrier surfaces, such as the epithelium of the lung and gut, blood brain barrier, and placenta (1), and at the liver (2). Type III interferons consist of three different functional genes in humans, *IFNL1*, *IFNL2*, *IFNL3*, and one pseudogene *IFNL4* (3–5). Mice have two functional genes, *Ifnl2* and *Ifnl3*, and pseudogene *Ifnl*1. Type III interferons are closely related to type I IFN, signaling through common Janus Kinase and Signal Transducer and Activator of Transcription (JAK-STAT) pathways that lead to transcription of IFN-stimulated genes (ISGs) (1, 6, 7). Specifically, type I IFN binds to the IFNαβ receptor (IFNAR) and type III IFN binds to the heterodimeric receptor (IFNLR), which is comprised of two subunits, IFNLR1 and interleukin (IL)10Rβ (3, 4). Binding of these ligands to their receptors leads to downstream phosphorylation of Signal Transducer and Activator of Transcription (STAT)1 and STAT2, subsequent recruitment of interferon regulatory factor (IRF)9, and transcription of interferon-stimulated genes (ISGs) (3, 8). Unlike type I IFN, however, type III IFN do not upregulate IRF1 that leads to downstream production of pro-inflammatory cytokines (9).

Despite similarities in downstream signaling between type I and type III IFN, they differ in cellular expression of their receptors. Since IFNAR is ubiquitously expressed, type I IFN-inhibition of viral replication occurs in many cell types. In contrast, IFNLR-mediated antiviral responses exhibit specificity for viruses that replicate at barrier surfaces due to its cell-specific expression (1, 10). In both mice and humans, IFNLR is expressed by epithelial cells (10), endothelial cells of the blood-brain barrier (11), macrophages (12–15), subsets of DCs (16–20), and neutrophils (21, 22). In humans, B cells have also been shown to respond to IFNL (23), while data regarding T cell responsiveness is inconclusive. The limited range of expression of IFNLR1 to mucosal surfaces and specific immune cells offers large potential for type III IFN to be used as therapeutic targets, given their higher tissue specificity and lower likelihood for off target effects, when compared to type I IFN.

In addition to differences in receptor expression, type I and type III IFN have different kinetics. Following hepatitis C infection of cultured cells, the type I IFN response is rapidly induced, while the type III IFN is slower and remains sustained for a longer duration (24, 25); similar kinetic differences are observed following aspergillus fumigatus infection in mice as well (22). Furthermore, treatment of human hepatocytes with type III IFN leads to a delayed and slow induction of ISGs, such as *ISG15*, interferon-induced GTP-binding protein *MX1*, and 2’-5’-oligoadenylate synthetase (*OAS1*), compared to type I IFN which leads to a faster and transient induction (9). These kinetic differences suggest that type I IFN may play a significant role early during acute infection, while type III IFN may promote long term control. Overall, the differences in cellular targets, kinetics of transcriptional effects, and immunomodulatory effects distinguish the effects of type I and III IFNs, which have led to studies examining type III IFN effects on immune cells in non-infectious contexts, including cancers and autoimmune diseases. Here we review the effects of type III IFN on leukocytes, certain cancers, and autoimmune diseases.

## Immune Modulation

### T Cell-Dendritic Cells Axis for IFNL Responses

**Figure 1:**
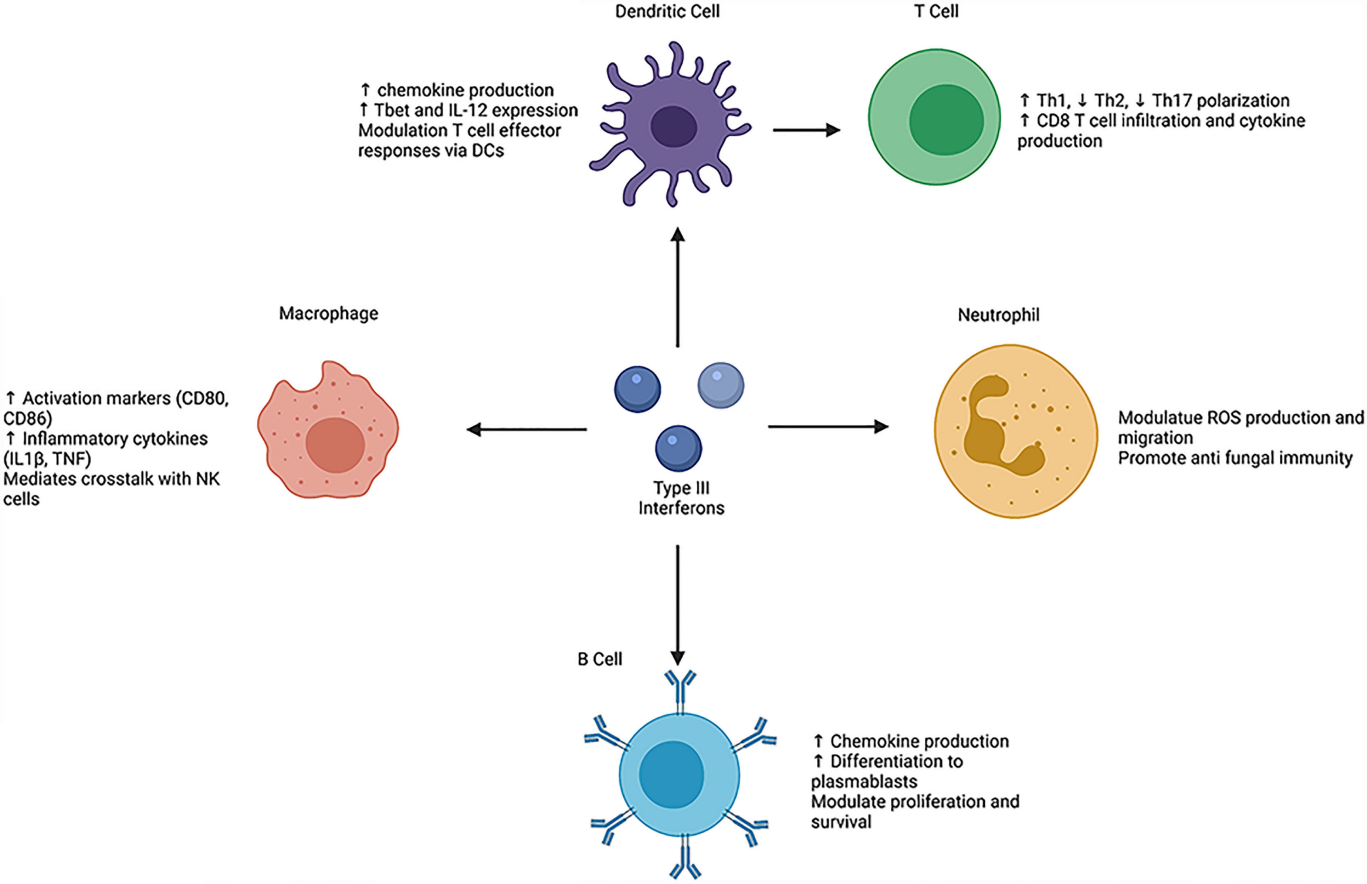
Schematic overview of the direct and indirect effects of type III interferons on leukocyte phenotypes and functions. helper T cell (Th), T-box transcription factor (Tbet), interleukin (IL), tumor necrosis factor (TNF), natural killer (NK), reactive oxygen species (ROS). Created with BioRender.com.

Many studies have suggested an important role for type III IFN in T cell polarization, with most providing evidence that IFNL down-regulates Th2 polarization and sustains Th1 activation (17, 26–30) (**Figure 1**). Intranasal, therapeutic administration of IFNL2/3 in a murine model of Th2-driven allergic asthma mitigated lung pathology and diminished levels of epithelial secreted Th2 cytokines, thymic stromal lymphopoietin (TSLP) and IL-33 in the bronchoalveolar lavage fluid (26). Additionally, adenovirus mediated expression of human IFNL1 in a murine asthma model led to attenuated eosinophilia, diminished antigen specific Th2 responses, and promotion of regulatory T (Treg) responses (31). IFNLs may also influence Th17 polarization, as treatment with IFNL2 reduced numbers of Th17 and γδIL-17+ cells in the inflamed joint compared to vehicle treatment in a model of collagen induced arthritis (32) and suppressed T cell IL-17 secretion in mediastinal lymph nodes of mice with allergic airway disease compared with vehicle treatment (17).

Studies of IFNL-mediated modulation of human immune cells have demonstrated similar Th1 promoting phenotypes. *In vitro* treatment of human peripheral blood mononuclear cells (PBMCs) with increasing concentration of IFNL1 increased production of the Th1 cytokine IFNγ and decreased production of Th2 cytokines interleukin (IL)-13, IL-4, and IL-5, with IL-13 expression being most consistently diminished following IFNL1 treatment (27, 28, 33). Treatment of human breast tumor suspensions with IFNL1 also induced expression of IL-12p40, a key driver of Th1 polarization (34). Effects of IFNLs on T cells may also be temporally dependent. In a model of murine lymphocytic choriomeningitis virus (LCMV) infection, IFNL dampened acute T cell responses, but promoted chronic T cell responses (35).

Given that IFNL has robust effects on T cell responses, it raises the question of whether IFNL acts directly or indirectly on T cells. Studies suggest that T cells do not respond to IFNL stimulation. T cells express low levels of *Ifnrl1* mRNA (21). *In vitro* treatment of T cells with IFNL failed to induce downstream phosphorylation of STAT1 or STAT3 (36) or expression of ISGs, such as *CXCL10*, *ISG15*, and protein kinase R (*EIF2AK2*) (20). Data instead suggest that IFNL may act on DCs to modulate and augment downstream T cell polarization and effector function (17, 18). IFNL2 treatment of lung and bone marrow derived CD11c^+^ DCs increased expression of both *Tbet* and *IL-12* (17). Furthermore, co-culture of IFNL2 treated DCs and ovalbumin (OVA) peptide-specific CD4^+^ T cells led to increased production of IFNγ and diminished production of IL-5, IL-13, and IL-17 (17). Transfer of these IFNL2 treated DCs to animals prior to OVA challenge skewed peripheral lymph node immune responses towards a Th1 phenotype and subsequently suppressed allergic airway disease (17). In a murine model of influenza A viral (IAV) infection *Ifnlr1* mRNA expression on DCs increased following infection, which correlated with increased IFNγ and tumor necrosis factor (TNF) production by lung CD8^+^ T cells. Conditional deletion of *Ifnlr1* on CD11c^+^ DCs in this model decreased numbers of infiltrating CD8^+^ T cells in the lungs (18). Comparison of wild-type (WT) and *Ifnlr1^-/-^* DCs also showed functional differences, as *Ifnlr1^-/-^* DCs showed diminished CD40 expression compared to WT DCs and ability to uptake and process antigen (18). Type III interferon mediated modulation of human DCs shows both similarities and differences compared to murine DCs. *In vitro* treatment of human peripheral DCs with IFNL increased MHC class I and class II expression, but did not have strong effects on CD80 or CD40 (19). Analysis of mixed lymphocyte reactions of human monocyte derived DCs (mDCs) with naïve T cells under various T cell polarizing conditions led to reduced IL-13 expression upon addition of IFNL (28). Another study of IFNL treated DCs co-cultured with CD4^+^ T cells showed increased proliferation of CD4^+^CD25^+^FoxP3^+^ Treg cells (37). Together, these data suggest that DCs are key mediators of IFNL driven T cell responses.

In addition to responding to IFNL, conventional DCs (cDC) can also produce IFNL (34). Following administration of polyinosinic:polycytidylic acid (polyI:C), murine CD8a^+^ cDCs and human CD141^+^ (BDCA3^+^) DCs produce significant quantities of IFNL1; in mice this depended on activation of toll-like receptors (TLR)3, IRF3, IRF7, and IRF8 (38). In corroboration, co-culture of a hepatitis C infected hepatoma cell line with human PBMCs increased IFNL production by CD141^+^ (BDCA3^+^) DCs (39). In a non-viral model, Hubert et al. demonstrated that human breast tumor associated cDCs upregulate IFNL1 gene and protein expression compared to adjacent normal tissue (34). Similar to cDCs, human plasmacytoid DCs (pDCs) can both produce and respond to IFNL. pDCs produce IFNL in response to herpes simplex virus, influenza virus, sendai virus, and HIV-1. Treatment of PBMCs with IFNL led specifically to upregulation of MHC class I and CD83 on pDCs but not other immune cell types, suggesting IFNL can act in an autocrine manner (16).

Aside from modifying DC mediated T cell activation and co-stimulation, IFNL may modulate chemokine driven migration and tissue specific infiltration of T cells. It has been established that type II IFN (IFNγ) induces downstream release of CXCL9, CXCL10, and CXCL11 (40, 41), chemoattractants responsible for guiding lymphocyte migration. Type III IFN treatment of human peripheral blood mononuclear cells also elevated expression of CXCL9, CXCL10, and CXCL11, independently of type II IFN signaling (42). CXCL10, in particular, is a key chemokine that recruits T cells to sites of inflammation and maintains their Th1 cell effector function, as the CXCL10 receptor, CXCR3, is preferentially expressed on Th1 cells compared to Th2 cells (40, 43, 44). These data suggest one mechanism by which IFNL treatment of cells promotes Th1 cell recruitment and maintenance. Recently, Goel et al. confirmed that type III IFN can upregulate CXCL10, as treatment of an immortalized human keratinocyte cell line with IFNL1 for 24 hours increased CXCL10 production compared with both vehicle and IFNα treatments. Additionally, in this model, IFNL1 treatment did not significantly increase CXCL9 levels compared to vehicle, and both IFNL1 and IFNα increased CXCL11 levels to a similar extent (45). In human breast tumors, Hubert et al. showed that cytokine analysis of the soluble tumor milieu demonstrated correlations between IFNL1 expression and CXCL10, CXLC11, and CXCL9, in addition to TNF and IL-12p40. Treatment of human breast tumor suspensions with IFNL1 resulted in increased expression of CXCL10 and CXCL11 (34). In contrast, treatment of immortalized human hepatocytes with IFNL3 failed to induce CXCL10 gene or protein expression (9). These data suggest that IFNL does not induce the same chemokine response in all cells, and instead promotes cell-specific chemokine responses. These differences may arise from the activation of non-canonical signaling pathways in certain cell types.

### Macrophage – NK Cell Axis

IFNL also alters human macrophage phenotypes, as fully differentiated macrophages derived from monocytes gain expression and functional capacity of the IFNLR1 receptor (12, 15) (**Figure 1**). *In vitro* studies show that macrophage treatment with all IFNLs inhibits replication of human immunodeficiency virus type I (HIV-1) *via* induction of JAK-STAT signaling (13, 46). Following IFNL3 treatment, macrophages upregulate ISG15, immune cell activation proteins, such as CD80 and CD86, and inflammatory cytokine production such as IL1β and TNF (12, 14). When treated with IFNL1 in the presence of a TLR7/8 agonist or lipopolysaccharide (LPS), human monocyte derived macrophages also increase production of IL-12p40; these effects are not observed upon treatment with IFNα and are thus specific to IFNL (14, 15). This increased IL-12 drives IFNγ production by natural killer (NK) cells. Incubation of NK cells with supernatants from macrophages treated with IFNL, LPS, and IFNγ increased NK cell IFNγ production compared with IFNα, LPS and IFNγ treatment (15). Wang et al. showed further evidence of IFNL-mediated macrophage NK cell crosstalk. During murine influenza A (IAV) infection, IFNL exerted antiviral activity by increasing NK cell numbers and promoting NK maturation. However, depletion of macrophages in this model reversed the effects of IFNL on NK cells (47). Beyond these studies, the ability of IFNL to promote NK cell-macrophages interaction is not well understood and requires further investigation in autoimmune diseases and cancer.

### B Lymphocyte Responses to IFNL

Human naïve and memory B cells express IFNLR1 (20, 48) and induce STAT1 phosphorylation following treatment with IFNL, suggesting that B cells are functionally responsive to IFNL (23) (**Figure 1**). B cell signaling occurs through the JAK-STAT pathway, as treatment with a JAK inhibitor prevented phosphorylation of STAT1 (23). B cell stimulation with IFNL and TLR7/8 agonist led to upregulation of only CD69, and no change in other co-stimulatory molecules, such as CD40, CD80, and CD86 (48). IFNL treatment of B cells also upregulated production of chemokines such as CXCL9, CXCL10, and CXCL11. Addition of IFNL following B cell receptor (BCR) stimulation upregulated the mechanistic target of rapamycin complex 1 (mTORC1) signaling pathway compared to BCR stimulation alone and promoted transcription of genes involved in differentiation of naïve B cells to plasmablasts (23). IFNL3 pretreatment of B cells (derived from healthy individuals) prior to stimulation with H1N1 reduced their proliferative capacity and IgG production (29). Novak et al. showed that a multiple myeloma cell line was responsive to IFNL, as IFNL1 treatment increased cell proliferation and promoted cell survival (49). In contrast to human B cells, murine B cells express low levels of *Ifnlr1* mRNA as measured by quantitative PCR and do not upregulate downstream ISGs in response to IFNL (21).

### Functional Regulation of Neutrophils by IFNL

IFNL binds to IFNLR on both murine and human neutrophils to alter their function (**Figure 1**). In *in vitro* studies, IFNL treatment of bone-marrow-derived neutrophils following stimulation with LPS or TNF resulted in diminished release of reactive oxygen species (ROS) and impaired degranulation (21). *In vivo* administration of IFNL diminished neutrophil secretion of IL-1β (32). IFNL also limits neutrophil migration, as IFNL2 treatment diminished neutrophil infiltration into the site of inflammation in a model of collagen induced arthritis (32). In a murine model of Aspergillus fumigatus infection, neutrophils upregulate IFNLR1 expression and type III IFNs promote their ROS generation (22).

## Autoimmune Diseases

Recent studies highlight that IFNL plays a significant role in immune-driven diseases; these diseases will be discussed in detail in the subsequent sections.

### Systemic and Cutaneous Lupus Erythematosus

Systemic lupus erythematosus (SLE) is an autoimmune and inflammatory disease that affects a number of organ systems including the skin, kidneys, and brain (50). In SLE there is increased activation of B cells by B cell activating factor (BAFF), TNFα, IL-6, and IL-21, in addition to increased production of autoantibodies by plasma cells. For this reason, initial SLE therapeutics, such as belimumab (anti BAFF), were targeted towards B cells (51, 52). Recently, other therapeutic targets have also been explored. For example, a recent phase 3 clinical trial demonstrated efficacy of anifrolumab, which targets the type I IFN receptor, in reducing SLE severity (53). The role of IFNL in SLE has been recently discussed in detail (54), and will be reviewed briefly here. Wu et al. showed that SLE patients had increased serum levels of IFNL compared to healthy controls and those with active disease had higher serum levels of IFNL compared to those with inactive disease (55). On the other hand, a study by Lin et al. did not observe a significant difference in IFNL levels between SLE patients and healthy controls. This discrepancy could be due to differences in limits of detection between the two studies. The study by Lin et al. had a larger percentage of serum values that fell below the limit of detection in healthy controls compared to SLE patients (56). Further analysis showed that SLE patients with renal or arthritis disease complications had increased serum IFNL levels compared to those without involvement of those organs (55, 57). In corroboration, single nucleotide polymorphisms (SNPs) at the IFNL gene locus have been correlated with risk of lupus nephritis (58, 59). Patients with active cutaneous lupus erythematosus (CLE) also have increased IFNL levels in their serum and skin lesions compared with healthy controls. Poly : IC exposure of epidermal explants from patients stimulated skin keratinocytes to further produce IFNL (60). Murine studies further highlight the ability of IFNL to promote SLE pathogenesis and suggest potential mechanisms. In a murine model of TLR7-induced lupus, IFNL promoted immune dysregulation through expansion of myeloid and T cell populations in the spleen and blood and induction of chemokine production by keratinocytes. Levels of serum IFNL and pDC derived IFNL were increased in mice following disease induction. Loss of IFNLR alleviated lupus induced splenomegaly and decreased numbers of splenic neutrophils, DCs, monocytes, CD4^+^ T cells and CD8^+^ T cells compared to WT animals. Analysis of murine skin revealed diminished inflammation, as *Ifnlr1^-/-^* animals had fewer infiltrating T cells, B cells, macrophages and neutrophils compared to WT animals. Lupus induced renal pathology was also significantly alleviated in *Ifnlr1^-/-^* mice compared to WT animals (45). Together, these data suggest that type III interferons may promote autoimmune disease and pathology in the contexts of systemic and cutaneous lupus erythematosus.

### Arthritis

In rheumatoid arthritis (RA) overactivation of multiple inflammatory pathways leads to subsequent inflammation of the synovium and cellular damage within joints (61). IFNL protein levels are upregulated in the sera of patients with rheumatoid arthritis compared with healthy controls (62–65); more specifically IFNL1 and IFNL2 levels are upregulated in patients with active disease (64). Disease severity in RA patients can be correlated with the presence of anti-cyclic citrullinated peptide (anti-CCP) antibodies (66). Analysis of IFNL levels in patients with and without anti-CCP antibodies showed increased IFNL1 in RA patients with detectable anti-CCP antibodies compared to RA patients negative for anti-CCP antibodies and healthy controls (65). This suggests a correlation between IFNL and disease activity in anti CCP antibody positive RA patients (62). Real time PCR analysis revealed increased expression of the receptor, *IFNLR1*, on PBMCs in RA patients compared with healthy controls (62). Treatment of a RA fibroblast cell line with IFNL1 upregulated cytokines IL-6, IL-8, and MMP-3 and downregulated the cytokine IL-10 (62). IFNL may also contribute to inflammation and cartilage degradation during osteoarthritis (OA) (67). OA patients had significantly increased serum IFNL1 levels and PBMC expression of *IFNLR1* compared to healthy controls (67). Similarly to RA, treatment of OA fibroblasts with IFNL1 induced expression of IL-1β, IL-6, IL-8, and MMP-3. In contrast, in a mouse model of rheumatoid arthritis, treatment with IFNL2 prevented arthritis progression, resolved inflammation, and improved pathology in comparison to a vehicle treated group (32). These data suggest isorform-specific IFNL may differentially impact inflammatory processes that mediate the pathogenesis of both OA and RA.

### Allergic Airway Disease

Allergic airway diseases are characterized by chronic Th2 driven inflammation of the upper or lower airways, eosinophilic accumulation, and IgE production (68). Studies of allergic airway disease in mice reveal the ability of IFNL to inhibit Th2 polarization (26) and promote IFNγ production (17), indicating that IFNL may enhance Th1 function (69). As discussed previously, therapeutic treatment of mice with IFNL following induction of murine asthma resulted in improved lung pathology and decreased production of Th2 cytokines such as TSLP and IL-33 (26). Furthermore, *Ifnlr1^-/-^* animals had exacerbated allergic airway disease, increased Th2 responses and IgE levels compared to WT animals (17).

Studies in humans have shown variable correlations between type III interferons and asthma. At baseline, both children and adults with asthma have increased levels of IFNL2 in their sputum compared with healthy controls; asthmatic children also had increased levels of IFNL1 in their sputum (70). Another study demonstrated that adults with asthma had increased levels of IFNL1 compared with healthy controls and that these elevated IFNL1 levels correlated with presence of neutrophilia in the sputum (71). In contrast, primary bronchial epithelial cells and bronchoalveolar lavage cells isolated from asthmatic patients demonstrated diminished production of IFNL2/3 following infection with rhinovirus in comparison with cells from healthy control patients. The decreased production of IFNL2/3 correlated with increased viral loads in the epithelial cells isolated from asthmatic patients compared to healthy controls (72). Since asthma exacerbations are frequently instigated by viral infections (73), it is important to further clarify the role IFNs play in the context of anti-viral immunity and airway inflammation.

### Inflammation of the Gastrointestinal System

Patients with inflammatory bowel disease (IBD) and mice with colitis demonstrate increased levels of *IFNL* and *IFNLR1* compared with controls (74). Specifically, lamina propria and intestinal epithelial cells (IECs) in colon tissues of patients with IBD, and mice with colitis, exhibit increased expression of IL28 and IL28R, respectively, compared with controls. In addition, *in vitro* studies demonstrated that IL28 induces phosphorylation (activation) of STAT1 in IECs, leading to their proliferation in organoid culture. In a murine model of immune activation induced colitis (dextran sulfate sodium-induced colitis), IFNL appears to diminish intestinal inflammation, as *Ifnrl1^-/-^* animals developed greater overall disease scores (21, 75), had decreased colon length (76), and had increased expression of oxidative stress genes within the colon (21) when compared with WT animals. Together these data suggest IFNL controls IEC proliferation and suppresses pro-inflammatory immune cells during inflammatory bowel disease. Consistent with this, administration of IFNL to mice with graft-versus-host disease (GVHD) induced by bone marrow transplantation exhibited improved survival, reduced GVHD severity, and enhanced epithelial proliferation and ISC-derived organoid growth after BMT (77).

Genetic variations of IFNL genes have been strongly correlated with outcomes following viral hepatitis. In humans a single nucleotide polymorphism (SNP) of *IFNL3* was correlated with viral clearance following hepatitis C treatment with pegylated interferon alpha and ribovarin (78, 79). A SNP of *IFNL4* also correlated with hepatic inflammation and fibrosis from both viral and non alcoholic fatty liver disease (80).

### Central Nervous System Autoimmunity

Recent work has demonstrated a role for IFNL in the pathogenesis of central nervous system (CNS) autoimmune disease [preprint, (81)]. In the experimental autoimmune encephalomyelitis (EAE) model of multiple sclerosis (MS), *Ifnlr1^-/-^* animals demonstrated improved clinical disease course and decreased spinal cord axonal injury compared with WT animals. These phenotypes correlated with decreased numbers of Th1 cells, reduced production of proinflammatory cytokines such as IFNγ and GMCSF, and diminished activation of antigen presenting cells within the CNS of *Ifnlr1^-/-^* animals compared to WT animals. Therapeutic targeting of the IFNL receptor *via* antibody mediated neutralization also recapitulated the EAE recovery phenotype observed in *Ifnlr1^-/-^* animals. Notably, analysis of post-mortem CNS tissue showed increased expression of IFNL and its receptor in lesions of MS patients compared to normal appearing white matter from non MS controls [preprint, (81)].

## Concluding Remarks

Type III interferon-mediated responses during infections were previously believed to be fairly limited, depending largely on cell-specific expression of the IFNLR, which occurs largely by cells at endothelial and epithelial barriers. However, given the ubiquitous expression of this receptor on both innate and adaptive immune cells, IFNL has increasingly been shown to play a critical role in sculpting overall immune responses during infection and inflammation, promoting Th1 polarization of CD4+ T cells over Th2. This is especially relevant during certain autoimmune diseases, where IFNL may exert pro-inflammatory effects. In addition, low levels of expression of IFNL in patients that develop virus-mediated autoreactive diseases at epithelial barriers may underlie tissue damage due to initial lack of virologic control, resulting in autoantigen-mediated autoimmunity. Currently, there are no drugs that block IFNL, and, therefore, no clinical trials looking at IFNL as a target to treat autoimmune diseases. However, as previously reviewed (54) JAK1 and JAK2 inhibitors are being tested for treatment of SLE. These inhibitors are not specific for IFNLR signaling, as they also target IFNAR. Future studies will define the feasibility of targeting IFNL to prevent chronic inflammatory diseases.

## Author Contributions

SM wrote the first draft, performed data mining, generated the figure, and contributed to editing. RK conceived the topic and outline of the review, recruited SM to participate, supervised the preparation of the drafts and figure, wrote the abstract and conclusions, and performed final edits. All authors contributed to the article and approved the submitted version.

## Funding

This work was supported by a grant from the National Multiple Sclerosis Society (RG 1801-29766), and grants from the National Institutes of Health (R56AI147623, R35NS122310, R01NS104471, R01NS116788, and R01NS052632; all to RK, and F31 NS108629-01A1; to SM).

## Conflict of Interest

The authors declare that the research was conducted in the absence of any commercial or financial relationships that could be construed as a potential conflict of interest.

## Publisher’s Note

All claims expressed in this article are solely those of the authors and do not necessarily represent those of their affiliated organizations, or those of the publisher, the editors and the reviewers. Any product that may be evaluated in this article, or claim that may be made by its manufacturer, is not guaranteed or endorsed by the publisher.
